# Natural and revolutionary tumor-specific T-cell therapy

**DOI:** 10.1007/s13659-024-00472-w

**Published:** 2024-08-19

**Authors:** Zhi Dai, Xue-Meng Liu, Yun-li Zhao, Li-Xing Zhao, Xiao-Dong Luo

**Affiliations:** Yunnan Characteristic Plant Extraction Laboratory, Key Laboratory of Medicinal Chemistry for Natural Resource, Ministry of Education, Yunnan Key Laboratory of Research and Development for Natural Products, School of Pharmacy, School of Chemical Science and Technology, Yunnan University, Kunming, 650500 People’s Republic of China

**Keywords:** Natural T-cell therapy, Specific killing, MHC^+^ tumor, CD28 signal, Without virus, Safety

## Abstract

Recently the FDA conducted a risk investigation and labeled the **Boxed Warning** for all BCMA- and CD19-directed CAR-T cell therapy, so does it mean that the public must take risk of secondary cancer to receive cell therapy? Here, without lentivirus and professional antigen presenting cell application, a novel tumor-specific T-cell therapy was successfully developed only by co-culturing MHC^+^ cancer cells and Naïve-T cells under the CD28 co-stimulatory signals. These tumor-specific T-cells could be separated through cell size and abundantly produced from peripheral blood, and would spontaneously attack target cells that carrying the same tumor antigen while avoiding others in vitro test. Moreover, it markedly decreased 90% tumor nodules companying with greatly improving overall survival (76 days *vs* 30 days) after twice infusion back to mice. This work maximally avoided the risks of secondary cancer and non-specific killing, and might open a revolutionary beginning of natural tumor-specific T-cell therapy.

**Graphical Abstract:**

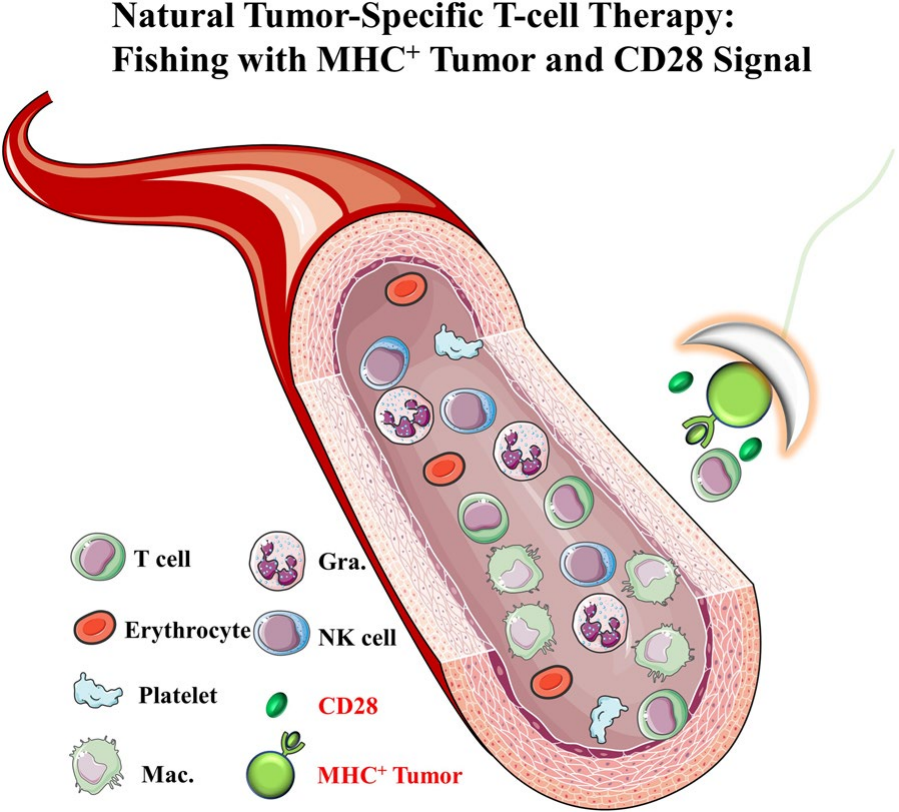

**Supplementary Information:**

The online version contains supplementary material available at 10.1007/s13659-024-00472-w.

## Highlights

1. Tumor specific T-cell generation without lentivirus and professional APC application.
2. Only by co-culturing MHC^+^ cancer cell and Naïve-T cell supplying CD28 signal.
3. Easy separation by cell size and fast generation from peripheral blood.
4. Maximally avoid troubles of viral safety and non-specific reaction.
5. Novel model of T-cell activation could be used to bioprospect medicinal molecules instead of CD28 from abundant natural products.

**Supplementary Information:**

The online version contains supplementary material available at 10.1007/s13659-024-00472-w.

## Introduction

In recent years, although the adoptive cell therapy (ACT) acquired practical success to treat cancers, especially the clinical success of the chimeric antigen receptors (CARs) T-cell therapy and the cognate T cell receptor (TCR-T) cell therapy, which becoming a landmark event [1, 2], its safety have been highly controversial. For example, the FDA announced that it begun to investigate the serious risk of secondary cancer following CAR-T treatment at November 28, 2023, and labeled the **Boxed Warning** for all BCMA- and CD19-directed CAR-T cell therapy at January 24, 2024. Besides, the on-target off-tumour toxicity and the cytokine release syndrome also happened frequently [3, 4]. Moreover, majority of the antigen-specific CARs or TCRs molecules currently in clinical use must be engineered by viral vectors, which greatly increased the risk of secondary cancer after T cells infusion [5, 6]. In addition, although the tumor-infiltrating lymphocyte (TIL) therapy is a virus-free ACT, it still has many troubles of non-specific killing [7]. For instance, the frequency of tumor antigen-specific T cells in tumors is often low so that majority of TILs are not the tumor-reactive lymphocytes [8]. Besides, the effective discovery and sorting the antigen-specific T cells from TILs were still full of troubles, despite pioneer works improving detection of the antigen-specific T cells in blood [9]. Moreover, combination of current traditional culture methods for TIL expansion in vitro have been shown to skew the ex vivo tumor-reactive lymphocytes repertoire, in which resulting in large number of the non-specific reactive lymphocytes also been infused back to patients [8, 9]. Therefore, clinical treatment and public’s safety require a virus-free and natural tumor-specific T-cell therapy that can be produced easily and quickly from peripheral blood rather than tumors.

## Results

### The design of a natural tumor-specific T-cell therapy

**Fig. 1:**
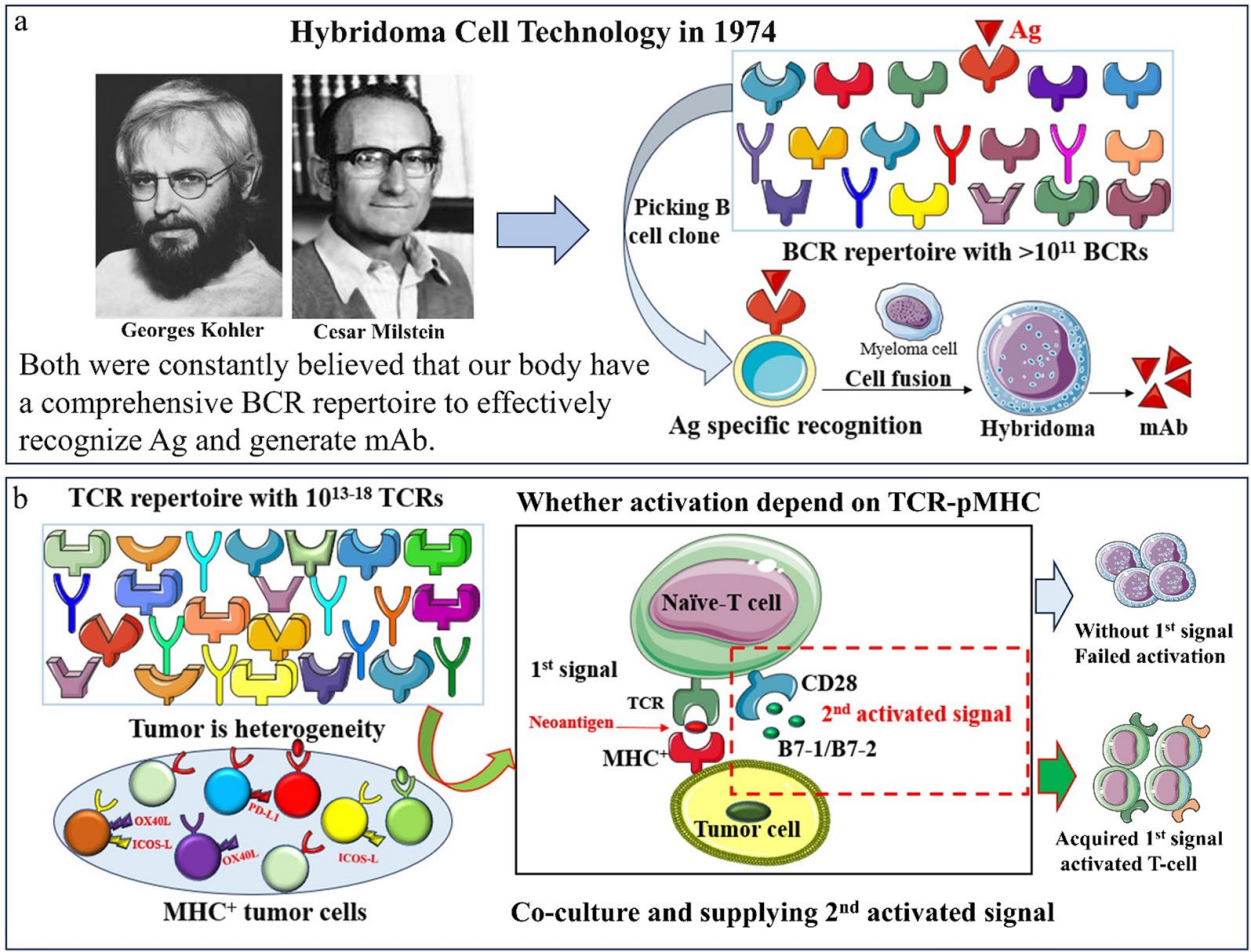
The generation and design of the natural tumor antigen-specific T-cell therapy. **a** The BCR repertoire and the hybridoma cell technology for Kohler G and Milstein C; **b** The TCR repertoire and the generation of this natural tumor antigen-specific T-cell therapy

The generation of this idea was inspired by the famous immunologists Kohler G and Milstein C. They believe that we have a comprehensive B cell receptor (BCR) repertoire with more than 10^11^ BCRs that can be used to recognize any foreign antigens and produce specific antibodies in the 1970s, which contributed the successful development of the hybridoma cell technology to produce monoclonal antibodies [10], and was awarded the Nobel Prize in 1984 (Fig. 1a). Even if 50 years have been gone, we are still inspired by their belief that our bodies possess an omnipotent antigen recognition receptor repertoire and the best recognition system to distinguish “self” and “non-self” to avoid against itself [11]. In fact, in addition to the BCR repertoire, our body also has a TCR repertoire for selecting specific T cells to clear infected or mutated cells. Inspired by this, we also firmly believe that as many as 10^18^ TCRs repertoire [12] can be used to recognize and clear target cells that containing any variant antigens, including tumor cells. Now, we developed a natural tumor antigen-specific T cell therapy by directly co-culturing MHC^+^ tumor cells and naïve-T cells supplying with CD28 co-stimulatory activation signals, in which it could be largely generated from peripheral blood without the application of virus and professional antigen presenting cell (APC) (Fig. 1b).

### The Naïve-T cells could be activated by MHC^+^*cancer* cells and CD28 signal

The activation of naïve-T cells must require both pMHC-TCR antigen signal and costimulatory activation signal [13], such as CD28 (CD28-B7-1/CD28-B7-2) signal. As we know, majority of tumors, including MHC^+^ and MHC^−^ tumors, escape the immune surveillance and killing with multiple mechanisms, such as hiding the tumor-specific antigen (TSA), and/or absence of costimulatory activation ligands, even over-expression immune checkpoint ligands (PD-L1, ICOS-L, OX40L etc.) [14]. However, due to the high heterogeneity of tumor tissues [15], there still may be existed a small number of tumor cells that can normally present pMHC antigen signals and do not have any suppression signals of immune checkpoints (Fig. 1b). Therefore, a few of naïve-T cells might be activated in a tumor antigen-specific manner and induce immune killing after receiving both these tumor cells pMHC antigen signals and supplementary CD28 co-stimulatory activation signals. As expected, testing of tumor sphering cells, generated from the MHC^+^ lung cancer cell (A549) [16] clonal formation, found that majority of tumor sphering cells were not induced immune killing and maintained nearly equal cell viability after supplementing with CD28 costimulatory signals compared to controls (Fig.S1a-b). Nevertheless, fortunately there were very small percentage of tumor cells that were carefully observed perforating killing (Fig.S1a) after supplementing with CD28 costimulatory signals. At the same time, significantly increased synthesis and secretion of perforin (PFP), granzyme B (GZMB) and IFN-γ were detected in the cell supernatant (Fig.S1c-i), in which suggested that a fraction of naïve-T cells was successfully activated and induced immune killing. This experiment proved that naïve-T cells can be directly activated by co-culture of MHC^+^ tumor cells and CD28 co-stimulatory signals, and the tumor-specific T-cells might be largely produced from peripheral blood without the use of virus, in which it completely avoided the shortcomings of viral safety and non-specific activation of current ACT therapy, and might become the most promising tumor-specific T cell therapy.

### The activated T-cell could be separated and sorted by cell size

**Fig. 2:**
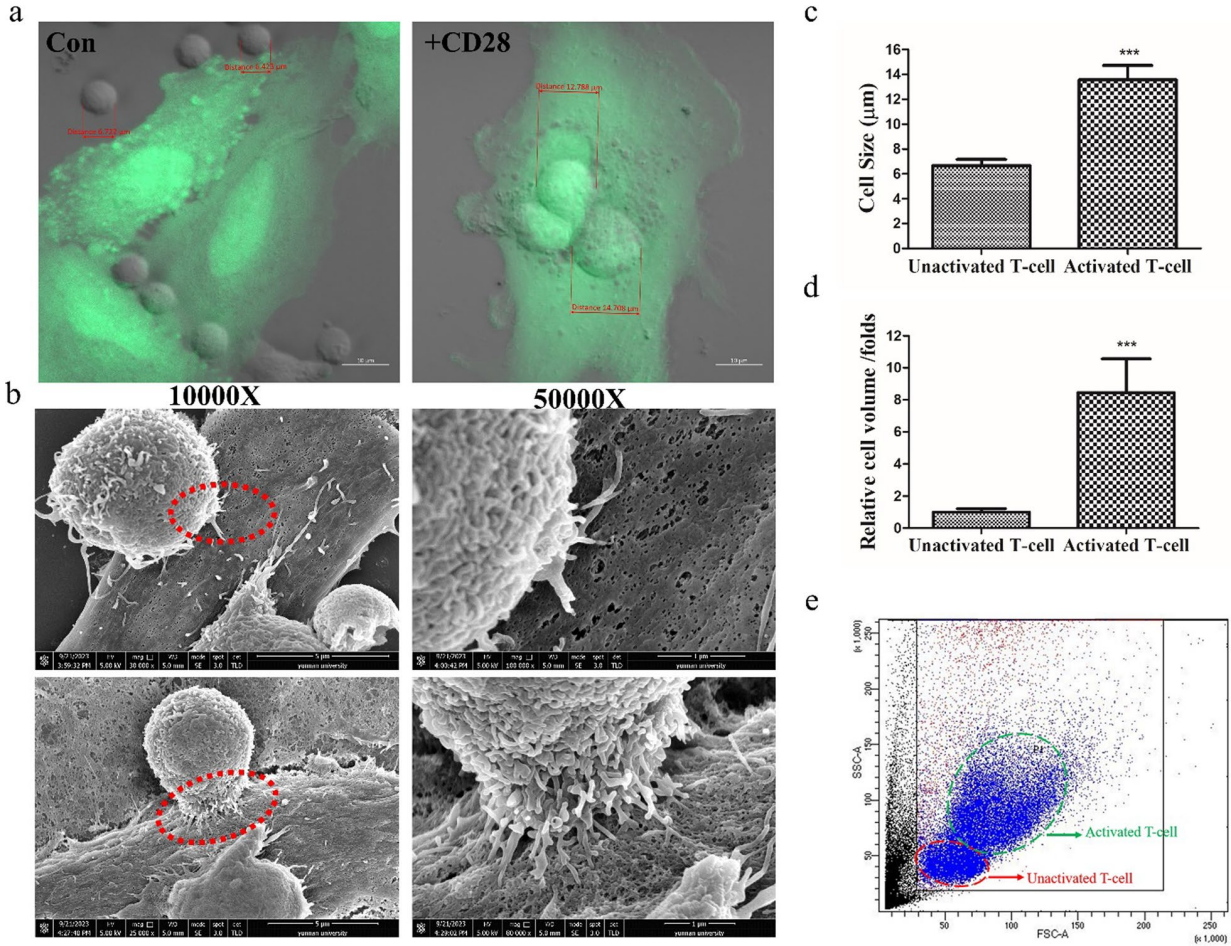
The characteristics of the tumor-specific activated T-cells. **a** The characteristics of unactivated T-cell (control) and activated T-cell (+ CD28); **b** The tumor cells were observed to closely bond to the activated T-cells, and were induced perforation killing through the scanning electron microscopic observation; **c**, **d** Representative quantitative analysis of cell size **c** and cell volume **d** between unactivated T-cell and activated T-cell. ***p < 0.001, t-test; **e** The separation and sorting of the activated T-cell by flow cytometry

In the above test, it was observed that all the T cells in the control group were loosely and randomly distributed around the tumor cells without any binding to them, and the cell size was between 5 and 7μm (Fig. 2a,c). While, after supplementing CD28 signal, it could be carefully observed that a few of tumor cells were surrounded by a fraction of T-cells and tightly bound to them, and induced perforated killing (Fig. 2a,b). Moreover, the cell size of these T cells was significantly increased to 10–14μm (Fig. 2a,c). This result could be supposed that these tumor-specific activated T-cells might be distinguished or separated from the large number of unactivated T-cells by cell size and cell volume (Fig. 2c,d), and fortunately it was further supported by the flow cytometry sorting (Fig. 2e and Fig.S2).

### The activated T-cell induced specific killing for target cells

**Fig. 3.**
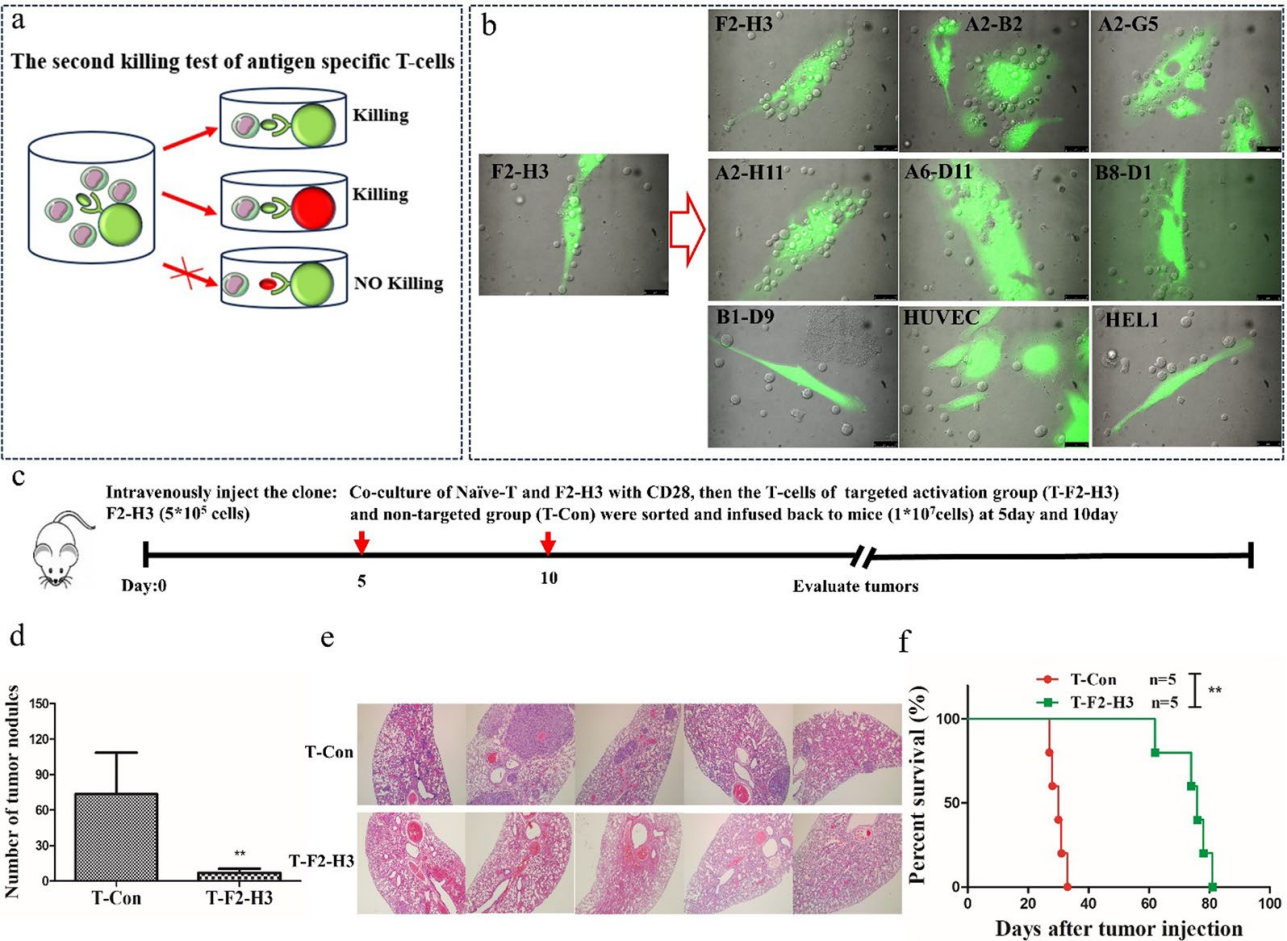
the natural tumor-specific T-cells could induce specific killing in vitro and vivo. **a** The treatment scheme of secondary killing test for tumor antigen-specific T-cells; **b** The secondary killing test of seven single clones and human umbilical vein endothelial cells (HUVEC) and human fetal lung fibroblast 1 (HEL1). It revealed that six clones (F2-H3, A2-B2, A2-G5, A2-H11, A6-D11 and B8-D1) could be induced secondary killing except for the one (B1-D9) and finite cell lines (HUVEC and HEL1); **c** The treatment scheme of specific killing in mouse; **d** Quantitation of tumor nodules at lung of targeted activation group (T-F2-H3) and non-targeted group (T-control). It revealed that T-F2-H3 treated mice showed less tumor burden compared with T-Con, in which the tumor nodules were cleared more than 90%,**p < 0.01, t-test. **e** the HE staining of both groups (T-con and T-F2-H3), which indicated that the tumor nodule of T-F2-H3 group was significantly less than the T-Con group. **f** the Kaplan–Meier analysis of mice survival, which revealled that the overall survival of T-F2-H3 was greatly extended compared with control cohort (median survival: 76 days *vs* 30 days, *p* = 0.0018)

As we know, the characteristic of precise antigen-specific killing is the most important feature of cellular immunity, which can maximize the avoidance of tissue and organ damage except for target cells, and it is also the principle that all the ACT therapies should be followed in future. Therefore, the tumor-specific killing was further evaluated through the secondary kill test. In this system, the antigen-specific activated T-cell can spontaneously search for, bind to, and attack target cells carrying the same antigen, while avoiding others (Fig. 3a). Firstly, there were seven single cell clones of A549 sphering cells picked by the limited dilution method, in which all the clones could be induced immune killing by a few of naïve-T cells after supplemented with CD28 costimulatory signals (Fig.S3). Then, the single cell clone (F2-H3) was used as the tumor specific signal to sensitize naïve-T cells, and subsequently these activated T-cells were obtained through flow cytometry sorting and subjected them to secondary killing test of 7 clones, including clone F2-H3. It showed that except for the single cell clone (B1-D9), six single cell clones, including F2-H3, were successfully induced perforation killing (Fig. 3b), in which appeared to indicate that all the six clones possessed the same antigen except for the one (B1-D9). In addition, this result was further demonstrated by secondary killing test of human umbilical vein endothelial cells (HUVEC) and human fetal lung fibroblast 1 (HEL1), both of which belong to finite cell line with no tumor antigens or proliferative mutations, and should not be induced killing by clone F2-H3-sensitized T-cells. As expected, both were not observed to induce killing (Fig. 3b).

### Infusion of tumor-specific T-cells could suppress tumors in mice

Next the in vivo antitumor effect was further examined in a heterotopic xenograft model using immune-deficient mice NOD.Cg-Prkdc^scid^IL2rg^tm1Sug^/JicCrl (NOG, seriously lack the B cells, T cells, NK cells, and therefore efficiently accept human cellular transplants [17]). The lung cancer cells (clone: F2-H3) were transplanted intravenously into mice (Fig. 3c). Then, the human peripheral blood T lymphocytes were co-cultured with cancer cells (F2-H3) supplying the CD28 signal for 48 h, and subsequently the T-cell repertoire was separated into targeted activation group (T-F2-H3) and non-targeted group (T-control) by flow cytometry sorting. On day 5, the T-cells of both groups were injected intravenously into transplanted mice after randomly grouping of treatment and control groups, respectively, and infused again at 10 day. Fortunately, the tumor nodules at lung were significantly decreased after infusion of F2-H3-activated T-cells (tumor nodules: 7 ± 2), whereas harbored more tumor nodules as observed in T-cells control group (tumor nodules: 73 ± 15) (Fig. 3d-e, Fig.S4), in which suggested that the tumor nodules were cleared more than 90% after twice infusion of T-cells targeted clone F2-H3. Besides, the mice overall survival of T-cells targeted clone F2-H3 was extended greatly compared with control cohort (median survival: 76 days *vs* 30 days, *p* = 0.0018) by the Kaplan–Meier analysis (Fig. 3f). Although the tumor burden was not completely eliminated and the overall survival was not completely rescued, this significant clear of tumor nodules in vivo was still exciting and successful in a model of heterotopic xenotransplantation against solid tumors by using natural T-cells. These results could be supposed that the overall survival might be further improved by increasing infusion frequency to clear residual tumor cells, and this work also might open a revolutionary beginning of safe and effective T-cell therapy without risk of secondary cancer.

## Discussion

Here we focused on the public requirements of current ACT and established a natural tumor-specific killing T-cells therapy without risk of secondary cancer, in which these tumor-specific killing T-cells could be generated fast and abundantly from peripheral blood without the professional antigen-presenting cell (APC) and virus modification. It might break a novel T-cells therapy field to extend the application of current ACT outside of CAR-T, TCR-T, TIL, and lymphokine activated killer cell (LAK) and cytokine induced killer cell (CIK) therapy. Moreover, the research might be attractive for more scientists of medicine, biology, structural biology, immunology, even chemistry to improve the technics or to reveal the tumor-specific antigens and structure of specific binding TCR, or to explore the mechanism of cancer metastasis and immune escape. Besides, compared with the biomacromolecules, such as CD28-B7-1/CD28-B7-2 signals, it would contribute to bioprospect less immunogenic and better penetrated natural molecules to break a novel field of natural products activated tumor-specific T-cell therapy (Nat-T cell therapy).

## Materials and methods

### Reagents and mice

The reagents used in T lymphocytes isolation were: RosetteSep™ Human T Cell Enrichment Cocktail (CAS#15021). The reagents for cytokines assay were used human ELISA kit: IL-1β (CAS: BMS224-2), IL-6 (CAS: EH2IL6), TNF-σ (CAS: KAC1751), IFN-γ (CAS: KHC4021), IL-12 (CAS: KHC0121), Perforin (PFP) (CAS: ab46068), Granzyme B (GZMB) (CAS: BMS2027). The CD28 functional monoclonal antibody (Invitrogen, CAS:16–0289-85). The mice used in investigation were the immune-deficient mouse NOD.Cg-Prkdc^scid^IL2rg^tm1Sug^/JicCrl (NOG) and purchased from the Beijing Vitalstar Biotechnology Co.,Ltd.

### Cell lines and cell culture

The lung cancer cell (A549), human umbilical vein vascular endothelial cells (HUVEC) and human fetal lung fibroblast 1 (HEL1) were harvested from ATCC, which had been authenticated and free of mycoplasma, and together with these single clones were cultured in DMEM completed medium (DMEM + 10% FBS + 1% P/S) at 37℃ 5%CO_2_ cell incubators.

### CD3^+^ total T lymphocytes isolation and cell culture

The CD3^+^ total naïve-T lymphocytes were isolated and purified directly from human peripheral whole blood by negative selection using the RosetteSep™ Human T Cell Enrichment Cocktail (CAS#15,021). The peripheral whole blood drawn into sodium heparin blood collection tubes and transferred to the 50 mL tube, then added the RosetteSep™ Human T Cell Enrichment Cocktail to the sample by 50μL/mL of sample. Mixed and incubated for 10 min at room temperature, then added equal volume recommended medium (PBS with 2%FBS) to dilute sample. Added 15 ml density gradient medium (CAS#07801) to the SepMate™ 50 tube (REF86450) and centrifuged it. Transferred the sample up to the density gradient medium gently and centrifuged it by 1200*g* for 10 min at room temperature. Poured supernatant into a new tube, then washed the enriched cells by recommended medium and centrifuged it by 300*g* for 10 min for twice. The enriched cells were the CD3^+^ T lymphocytes and cultured in DMEM 1640 + 10% FBS + 1% P/S + IL-2 (200U/ml) at 37℃ 5% CO_2_ cell incubator.

### Generation of tumor sphering cell by clonal formation

The tumor sphering cells of lung cancer cell A549 were harvested by the clonal formation assay. The A549 cells (2*10^4^cell/6-well-plate) were mixed in the 0.3% soft agar (1 ml), then put on the 0.8% soft agar (1 ml) and supplied with DMEM completed medium (2 ml), after 10 day, the sphering cells were harvested by the clonal picking.

### The generation of the green fluorescent protein (GFP) cell

The tumor sphering cells, together with HUVEC and HEL1 were infected with lentivirus that stable expressed green fluorescent protein (GFP) for twice. Then, most of these cells could be conveniently observed and distinguished in the co-culture system by the fluorescence microscope.

### Co-culture of tumor sphering cells and naïve-T cells

The tumor sphering cells of A549 (1*10^4^cells/24-well-plate) and naïve-T cells (2*10^5^cells) were mixed and put into 24-well-plate. Then, the CD28 functional monoclonal antibody was added at concentration of 100 ng/ml, and the same volume of PBS (PH7.2) acted as the control groups. The phenotypic observation was performed by the fluorescence microscope for 3-5 day. The cell viability assay of tumor cells was carried out by the MTS, and the supernatant medium was centrifuged and measured the effector cytokines.

### Measurement of the cytokines

The cytokines: IL-1β (CAS: BMS224-2), IL-6 (CAS: EH2IL6), TNF-α (CAS: KAC1751), IFN-γ (CAS: KHC4021), IL-12 (CAS: KHC0121), Perforin (PFP) (CAS: ab46068), Granzyme B (GZMB) (CAS: BMS2027) was measured according to the ELISA kit described method from the supernatant.

### Establishment of the single cell clone from tumor sphering cells

The single cell clones of A549 sphering cells were picked by the limited dilution method. The A549 sphering cells were limited diluted in medium (ratio: 1cell/well) and put into 96-well-plate, then, picked the single cell wells for further culture. At 15 day, these single cell clones were again limited diluted in medium (ratio: 1cell/well) and put into 96-well-plate, and choose the single cell wells for expansion.

### Observation of scanning *electron* microscopic

The clone (F2-H3) cells (1*10^4^cells/24-well-plate) together with naïve-T cells (2*10^5^cells) were mixed and put on conductive silicon wafer, in which the CD28 functional monoclonal antibody (100 ng/ml) and the PBS (PH7.2) was added, respectively, and cultured in 24-well-plate for 2 day. Then, the sections were fixed, dehydrated and dried as the demands of scanning electron microscopic for further observation.

### The flow cytometry sorting

The clone (F2-H3) cells (1*10^4^cells/24-well-plate) together with naïve-T cells (2*10^5^cells) were mixed and put into 24-well-plate, in which the CD28 functional monoclonal antibody (100 ng/ml) and the PBS (PH7.2) was added, respectively, and cultured for 2 day. Then, harvested all the cells for flow cytometry sorting. According to the GFP, the mix could be divided into GFP^+^ cells (survival cancer cell) and GFP^−^ cells (T-cells), then, the GFP^−^ cells further separated into activated T-cells and inactivated T-cells by cell size and cell volume.

### The secondary killing test

The single cell clone (F2-H3) (1*10^4^cells/24-well-plate) together with naïve-T cells (2*10^5^cells) were mixed and put into 24-well-plate, in which the CD28 functional monoclonal antibody (100 ng/ml) and the PBS (PH7.2) was added, respectively, and cultured for 2 day. Then, the activated T-cells were harvested by the flow cytometry sorting, and subsequently used to evaluate the secondary killing of 7 single clones (F2-H3, A2-B2, A2-G5, A2-H11, A6-D11, B8-D1, B1-D9) and HUVEC, HEL1 (1*10^4^cells/24-well-plate). The phenotypic observation was performed by the fluorescence microscope for 3 day.

### Anti-tumor evaluation of tumor-specific T-cells in vivo

The immune-deficient mouse NOD.Cg-Prkdc^scid^IL2rg^tm1Sug^/JicCrl (NOG) used to test the anti-cancer efficacy in vivo and purchased from the Beijing Vitalstar Biotechnology Co.,Ltd. At the day 0, the clone cells F2-H3 (5*10^5^ cells/mouse) was injected intravenously into the mice (8 weeks old, female). Then, the human peripheral blood T lymphocytes (1*10^7^ cells) were co-cultured with cancer cells (F2-H3) (5*10^4^cells/6-well-plate) supplying the CD28 signal for 48 h, and subsequently the T-cell repertoire was separated into activated group (T-F2-H3) and inactivated group (T- control) by flow cytometry sorting. Then, both group T-cells were further expanded proliferation in vitro supplied with Human T-Activator CD3/CD28 Dynabeads (CAS# 111.61D). At 5 day after engraftment, the T-cells (1*10^7^ cells/mouse, 0.2 ml physiological saline) of both groups were injected intravenously into transplanted mice, and infused again at 10 day. The mice survival, body weight and fur state were recorded and analyzed after treatment.

These peripheral blood samples (10-20 ml) were donated from one healthy donor, and the informed consent was obtained after the nature and possible consequences of the studies were explained. The used mice were in accordance with institutional guidelines of animals' care. We complied with all ethical standards of the Research Ethics Committee of Yunnan University. The sample was isolated and purified using RosetteSep™ Human T Cell Enrichment Cocktail, (CAS#15021), as previous described method.

### Statistics analysis

Differences between two groups were compared using Student’s or Welch’s t-test.

### Supplementary Information

Supplementary Material 1.

## Data Availability

All data are available in the main text or the supplementary information. Additional information and materials will be made available upon request.
