# Supplementary material for: Natural and revolutionary tumor-specific T-cell therapy
Source: Nat Prod Bioprospect. 2024 Aug 19;14(1):48. doi: 10.1007/s13659-024-00472-w (PMC11333775; doi:10.1007/s13659-024-00472-w)
Supplement: Supplementary file 1 — Supplementary Material 1. [file 13659_2024_472_MOESM1_ESM.docx]

**Supplementary information**

**Natural and Revolutionary Tumor-specific T-cell Therapy**

Zhi Dai^1^*, Xue-Meng Liu^1^, Yun-li Zhao^1^, Li-Xing Zhao^1^, Xiao-Dong Luo ^1^*

^1^ Yunnan Characteristic Plant Extraction Laboratory; Key Laboratory of Medicinal Chemistry for Natural Resource, Ministry of Education; Yunnan Key Laboratory of Research and Development for Natural Products; School of Pharmacy; School of Chemical Science and Technology, Yunnan University, Kunming, 650500, P. R. China.

*Corresponding author: Xiao-Dong Luo,Tel.: +86-871-65223177, E-mail: [xdluo@ynu.edu.cn](mailto:xdluo@ynu.edu.cn); Zhi Dai, E-mail: [daizhi@ynu.edu.cn](mailto:daizhi@ynu.edu.cn)

**Contents:**

Fig. S1. The phenotypic observation of lung cancer sphering cells and measurement of cytokines.

Fig. S2. The cell sorting of tumor-specific activated T-cells.

Fig. S3. The immune killing test of single cell clones.

Fig. S4. The tumor-specific killing in mice.


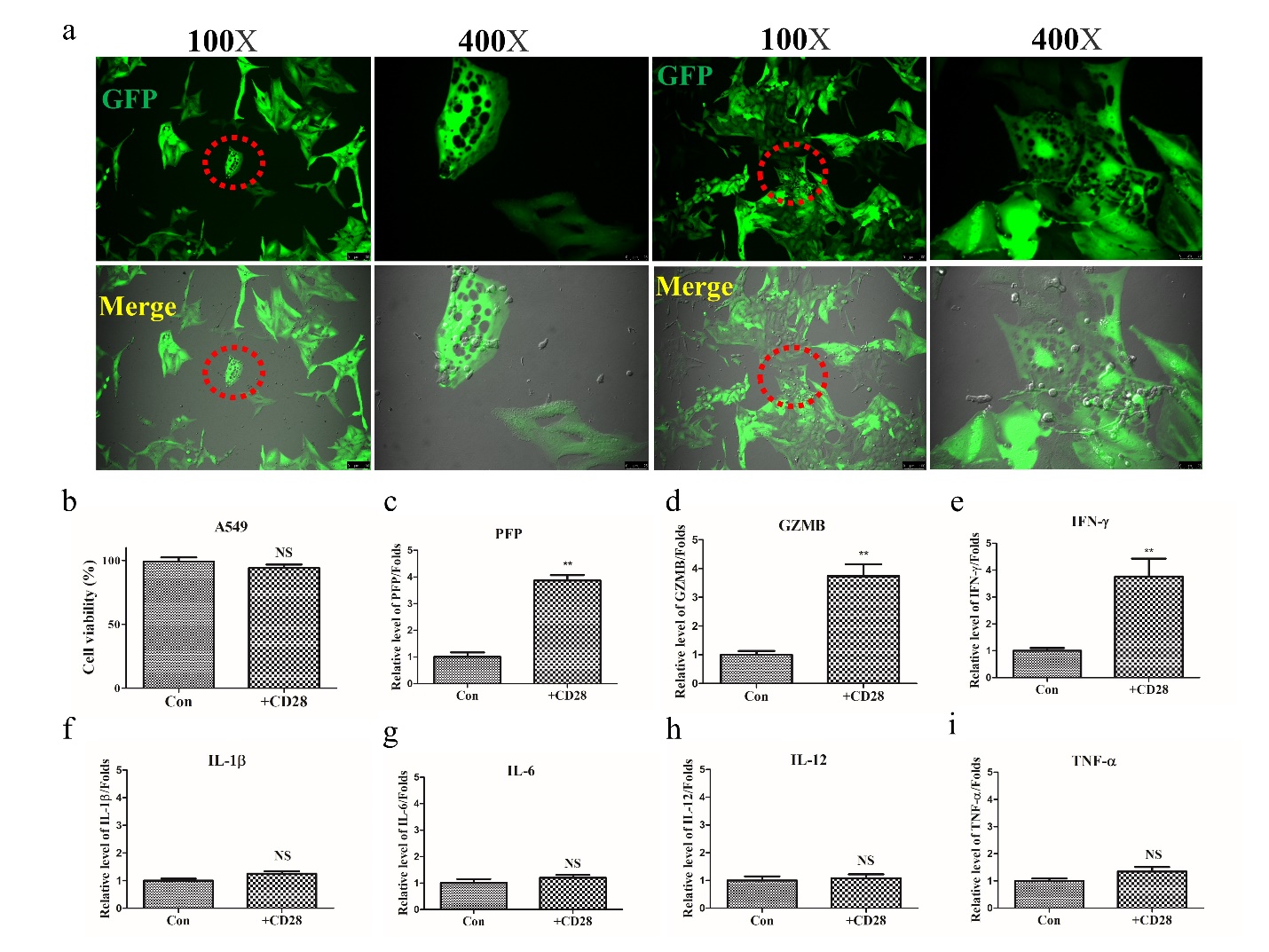
Fig. S1.

**The phenotypic observation of lung cancer sphering cells and measurement of cytokines.** (a) The phenotypic observation (100X and 400X) of lung cancer sphering cells (A549-Sphering-GFP) co-cultured with naïve-T cells after supplying the CD28 costimulatory signal; (b) the cell viability assay of lung cancer sphering cells between the control groups and supplying the CD28 groups; (c-i) Representative quantitative analysis of perforin (PFP), granzyme B (GZMB), IFN-γ, IL-1β, IL-6, IL-12 and TNF-α in the supernatant between the control groups and supplying the CD28 groups. **p<0.01, t-test.


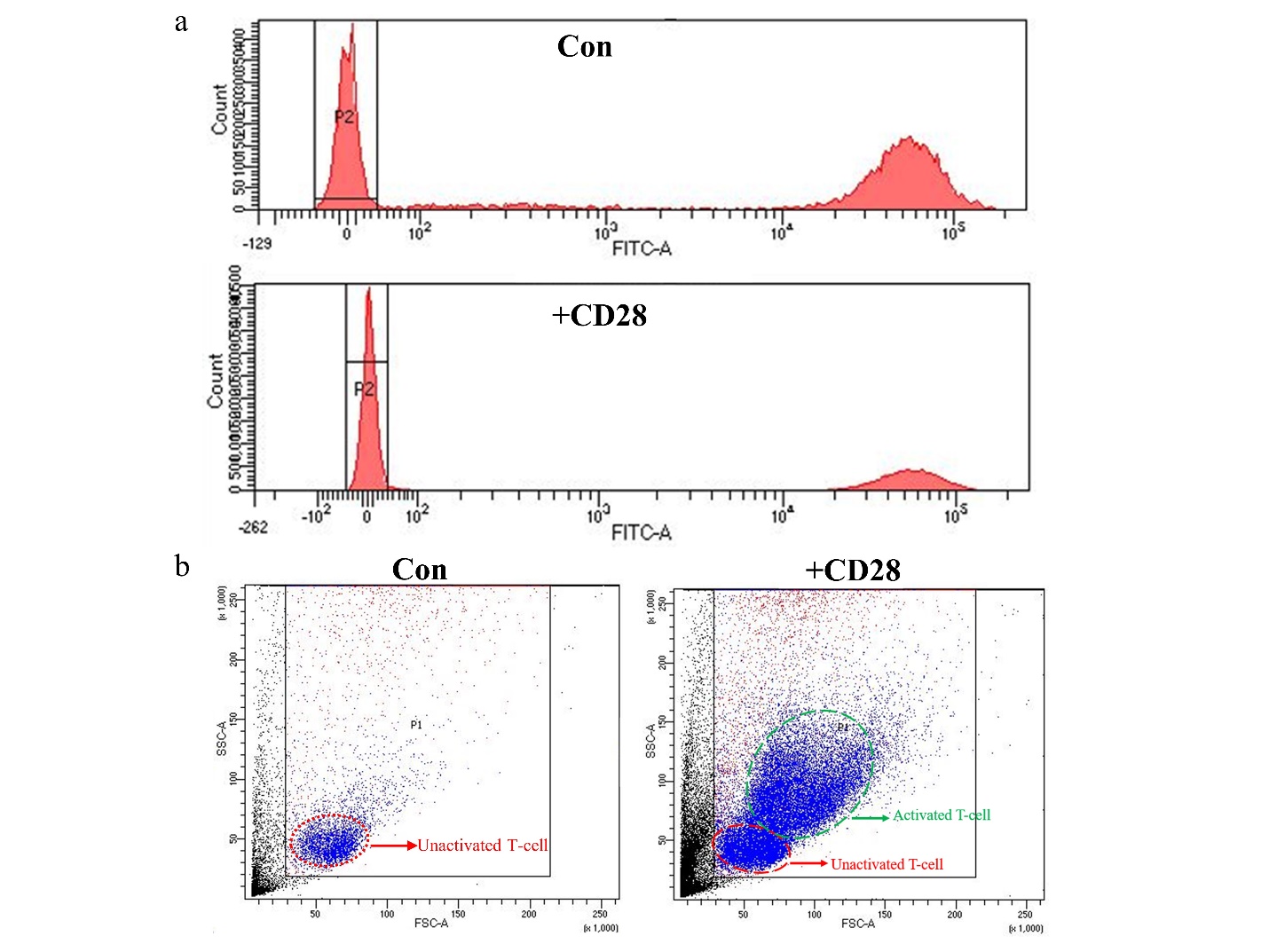
Fig. S2.

**The cell sorting of tumor-specific activated T-cells.** (a) the GFP^+^ cells (survival cancer cell) and GFP^-^ cells (T-cells) assay between the control groups and supplying the CD28 groups; (b) the activated T-cells and unactivated T-cells analysis among the GFP^-^ cells between the control groups and supplying the CD28 groups by the cell size and cell volume.


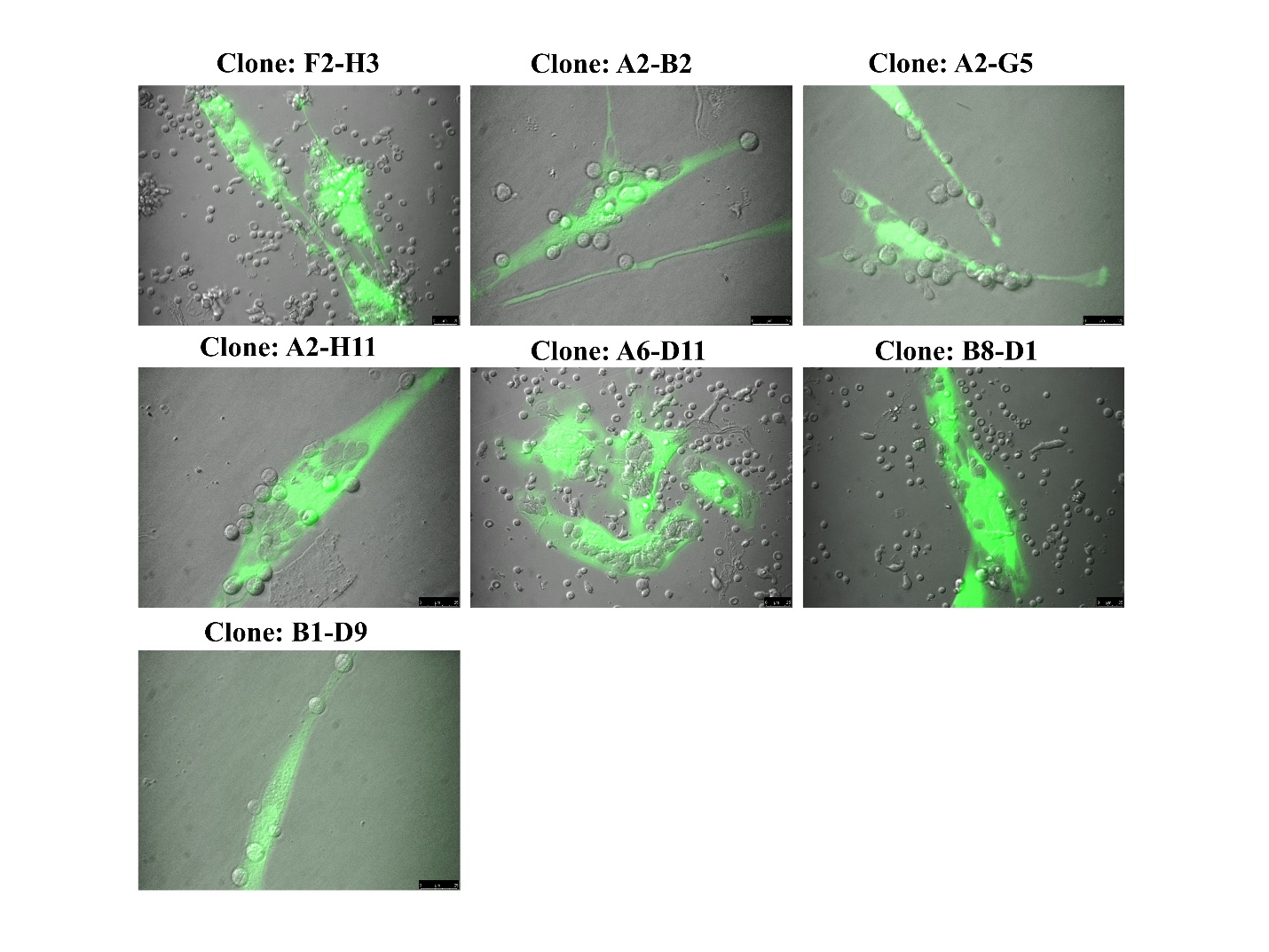
Fig. S3.

**The immune killing test of single cell clones.** The immune killing test and observation of seven clones that co-cultured with naïve-T cells accompanying with the CD28 costimulatory signal. It showed that these clones could be induced immune killing under the co-culture system, in which indicated that these clones could normally present pMHC antigen signal and used to generate tumor-specific T-cells.


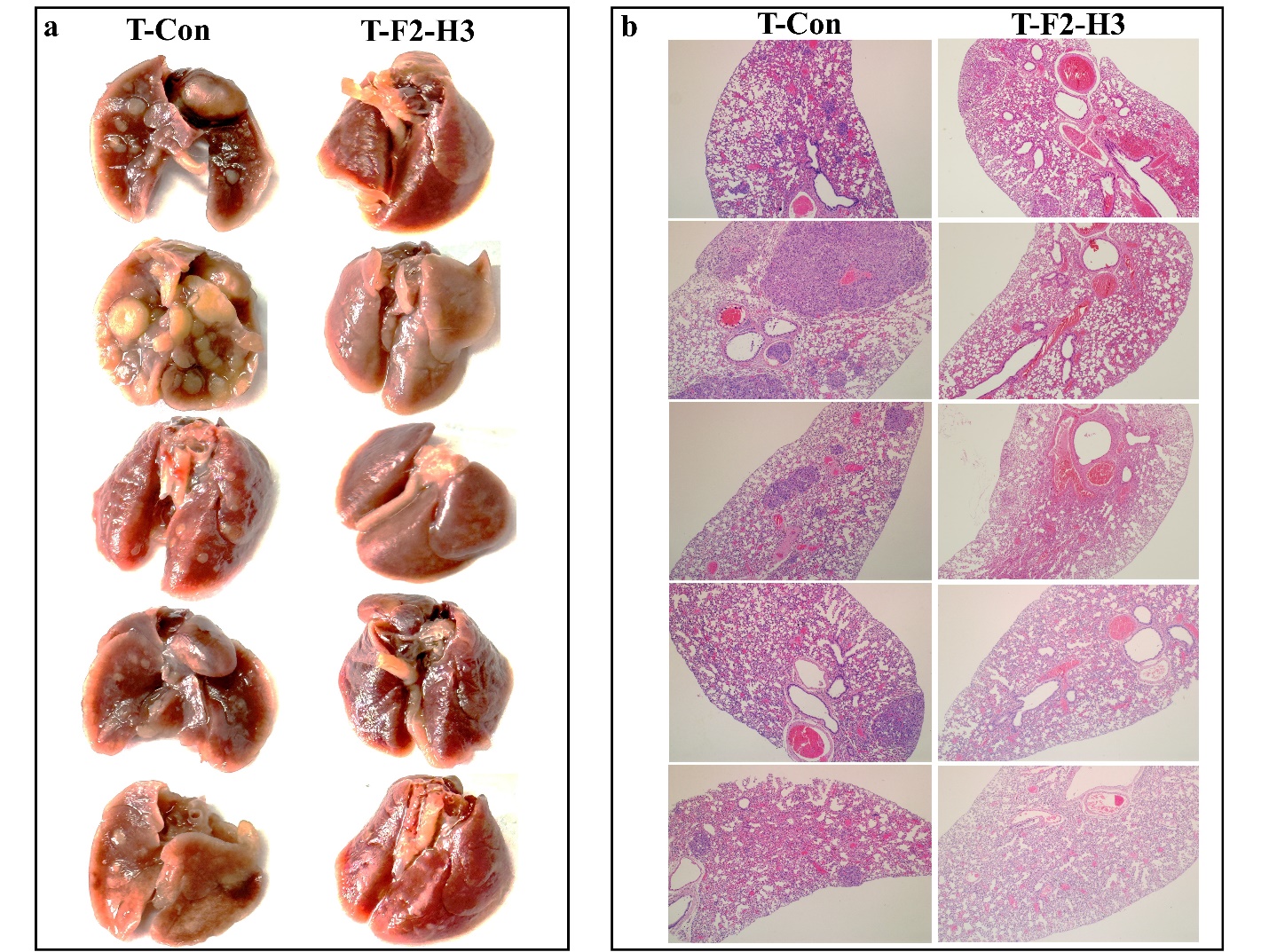


Fig. S4.

**The tumor-specific killing in mice.** (a) The phenotypic observation of tumor nodules at lung for T-Con and T-F2-H3 group, which indicated that the tumor nodules of T-F2-H3 were significantly less than the T-Con group. (b) The HE staining of both groups (T-con and T-F2-H3), which indicated that the tumor nodule of T-F2-H3 group was markedly decreased compared with the T-Con group.
